# Functional, motor, and sensory assessment instruments upon nerve repair in adult hands: systematic review of psychometric properties

**DOI:** 10.1186/s13643-018-0836-0

**Published:** 2018-10-27

**Authors:** Marisa de Cássia Registro Fonseca, Valéria Meireles Carril Elui, Emily Lalone, Natália Claro da Silva, Rafael Inácio Barbosa, Alexandre Márcio Marcolino, Flávia Pessoni Faleiros Macedo Ricci, Joy C. MacDermid

**Affiliations:** 1 Department of Health Sciences, Ribeirao Preto Medical School, University of Sao Paulo, Ribeirao Preto, SP Brazil; 2 Clinical Research, Hand and Upper Limb Centre, St Joseph’s Health Centre, Western University, London, ON Canada; 3 Rehabilitation and Performance Program, University of São Paulo, Ribeirão Preto, Brazil; 4 Laboratory of Assessment and Rehabilitation of Locomotor System (LARAL), Federal University of Santa Catarina, Araranguá, SC Brazil; 5 Rehabilitation Science, School of Rehabilitation, McMaster University, Hamilton, ON Canada

**Keywords:** Hand injuries, Validity of tests, Evaluation, Outcome, Systematic review

## Abstract

**Background:**

Outcome after nerve repair of the hand needs standardized psychometrically robust measures. We aimed to systematically review the psychometric properties of available functional, motor, and sensory assessment instruments after nerve repair.

**Methods:**

This systematic review of health measurement instruments searched databases from 1966 to 2017. Pairs of raters conducted data extraction and quality assessment using a structured tool for clinical measurement studies. Kappa correlation was used to define the agreement prior to consensus for individual items, and intraclass correlation coefficient (ICC) was used to assess reliability between raters. A narrative synthesis described quality and content of the evidence.

**Results:**

Sixteen studies were included for final critical appraisal scores. Kappa ranged from 0.31 to 0.82 and ICC was 0.81. Motor domain had manual muscle testing with Kappa from 0.72 to 0.93 and a dynamometer ICC reliability between 0.92 and 0.98. Sensory domain had touch threshold Semmes-Weinstein monofilaments (SWM) as the most responsive measure while two-point discrimination (2PD) was the least responsive (effect size 1.2 and 0.1). A stereognosis test, Shape and Texture Identification (STI), had Kappa test-retest reliability of 0.79 and inter-rater reliability of 0.61, with excellent sensibility and specificity. Manual tactile test had moderate to mild correlation with 2PD and SWM. Function domain presented Rosén-Lundborg score with Spearman correlations of 0.83 for total score. Patient-reported outcomes measurements had ICC of 0.85 and internal consistency from 0.88 to 0.96 with Patient-Rated Wrist and Hand Evaluation with higher score for reliability and Spearman correlation between 0.38 and 0.89 for validity.

**Conclusions:**

Few studies included nerve repair in their sample for the psychometric analysis of outcome measures, so moderate evidence could be confirmed. Manual muscle test and Rotterdam Intrinsic Hand Myometer dynamometer had excellent reliability but insufficient data on validity or responsiveness. Touch threshold testing was more responsive than 2PD test. The locognosia test and STI had limited but positive supporting data related to validity. Rosén-Lundborg score had emerging evidence of reliability and validity as a comprehensive outcome following nerve repair. Few questionnaires were considered reliable and valid to assess cold intolerance. There is no patient-reported outcome measurement following nerve repair that provides comprehensive assessment of symptoms and function by patient perspective.

**Electronic supplementary material:**

The online version of this article (10.1186/s13643-018-0836-0) contains supplementary material, which is available to authorized users.

## Background

Traumatic nerve injuries in the hand are common and can result in chronic dysfunction, extensive rehabilitation, and repeated surgeries. These injuries have a higher incidence in young males [1–3]. The combination of residual disability at a young age has a profound economic lifelong impact through the impacts on work ability. Additionally, impairment and disability after nerve injury result in reduced quality of life [4–6]. Previous studies have shown that, as the complexity of the case increases, the cost and duration of treatment also increases [2, 7, 8]. When the trauma to the hand is poorly managed either during surgery or rehabilitation, it is not only the patient, but also the whole family that suffers [9].

Patients with peripheral nerve injuries that affect the hand need specialized surgery and rehabilitation to regain function [10]. Nerve injuries can affect mobility and sensibility leading to cold sensitivity and pain [11]. A previous systematic review of nerve repair techniques determined that patient’s age, tension of repair, time of repair, level of injury, and scar formation following surgery affect the prognosis for functional outcome [12]. Nevertheless, some degree of persistent posttraumatic disability often persists after nerve laceration and repair [4]. Therefore, assessing patient with a valid outcome method or instruments following nerve repair is essential as it provides information about patients’ sensorimotor deficits and function [1, 13–16] and is critical to developing best evidence in repair and rehabilitation.

Since Tinel [17], many authors have described several approaches related to functional assessment after nerve lesion and repair [18–23]. These range from an ordinal “numerical grading system” based on a motor and sensory scale [18, 20] to a multi-dimensional comprehensive scale based on sensory, motor, and pain domains in a model instrument for documentation of outcome after nerve repair [24, 25], and a variety of other instruments [14, 16, 26–29].

Selecting an instrument or a battery of tests for assessment of nerve outcomes requires knowledge of the clinical measurement properties of the potential test options. Ideally, the selected measurement should be reliable and be able to distinguish functional outcomes, measure change in clinical status, and predict the outcome of different interventions [21, 23, 26, 30–32]. Based on principles of evidence-based practice [33–36], any outcome measurement [11, 31–33], including those for peripheral nerve repair assessment, it is essential to know the psychometric [37, 38] properties such as reliability, validity, and responsiveness [30, 38, 39]. Reliability is also referred to as reproducibility, stability, repeatability, variability, consistency, concordance, dependability, precision, and agreement [40–42]. It is the degree to which the measurement is free of error, depending on the specific measurement instrument, persons performing the measurement, patients, and circumstances under which the measurement is taken. A repeated measurement over time is called test-retest, by different persons on the same occasion is called inter-rater, or by the same persons on different occasions is called intra-rater. Reliability can be assessed over different intervals or raters and by a variety of different statistical methods [37, 42]. Validity is the measurement property that defines the extent to which an instrument measures the construct it aims to measure (truthfulness). There are a variety of types of validity reflecting the different purposes of clinical measurement and different techniques for assessing the extent to which a measure can fulfill these purposes. The main types of validity are *content validity* related to relevance and comprehensiveness, *criterion validit*y (concurrent and predictive) which relates to a *gold standard* or criterion referent measure, and *construct validity* (structural validity, discriminative, convergent, divergent and cross-cultural validity). A variety of statistical methods can be applied to assess these properties. Responsiveness (*longitudinal validity*) is a property of an instrument, which detects changes in the construct over time [42, 43], and is also has a range of statistical techniques designed to assess the extent and classification accuracy of change that occurs due to time or intervention.

Jerosch-Herold [44] published a systematic review focused just on sensory tests for nerve repair assessment in 2005, and at that time, there were few instruments with sufficient evidence related to the reliability, validity, and responsiveness of tests to assess sensibility after nerve repair [43, 45]. Since evidence accumulates over time and nerve outcome measures should extend beyond sensation, there was a need to conduct a broader and more current review of nerve repair outcome measures. The objective of this study was therefore to systematically review and summarize available evidence on the clinical measurement properties of instruments, which evaluate motor, sensory, and functional status after primary or secondary nerve repair in adult hands using a standardized critical appraisal tool of quality for psychometric articles and multiple independent appraisers.

## Methods

This is a systematic review of health measurement instruments. A literature search was conducted using the following databases: PubMed/MEDLINE, SCOPUS, Cochrane Library, PEDro, CINAHL, PsychInfo, EMBASE, SciELO, LILACS, SPORTDiscus, ERIC, and Google Scholar, ranging from 1966 to 2017. The search was limited to publications, written in English, Spanish, or Portuguese.

Keywords used included “hand” or “hand injuries” or “injury” and (“peripheral nerve injury” or “peripheral nerve repair” or “nerve repair” or “nerve injury”) and (“outcome” or “outcome assessment” or “assessment” or “instrument” or “tool” or “functional outcome” or “documentation” or “evaluation”) and (“reliability” or “responsiveness” or “validity” or “validation” or “psychometrics”) and (“clinical measurements” or “Rasch analysis” or “factor analysis” or “cross cultural translation”).

### Selection for inclusion

Studies were included if they addressed at least one psychometric property, related to motor impairment, sensory status, cold intolerance, pain, or functional status from primary or secondary nerve repair at any level on the forearm, hand, fingers, and/or wrist. These instruments included any device developed to measure motor and sensory function, but also cold intolerance or pain outcome or functional status.

Articles were excluded in our final review if they met any of the following criteria: descriptive, epidemiologic, or interventional studies; pediatric participants or adults with neuromuscular diseases or any other disorder of central nervous system or even generic outcomes to trauma not specifically related to nerve repair in the hand; and unpublished, conference proceeding, thesis, and dissertation and non-human studies.

**Fig. 1:**
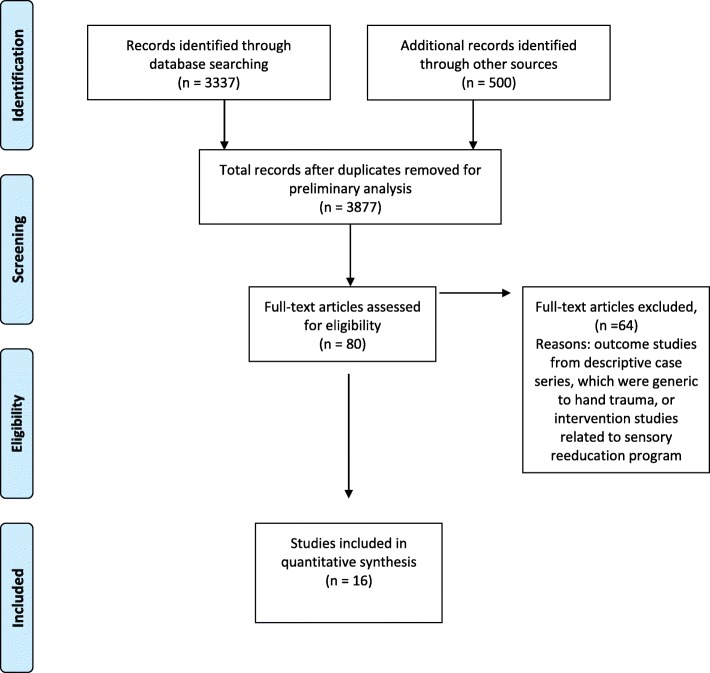
PRISMA flow chart for search strategy

The initial selection, based on titles and abstracts, was performed by one reviewer and reviewed by the second to identify any potentially relevant articles that have been missed in the original screening (Fig. 1). If there was any uncertainty of eligibility for inclusion, the full text was obtained for the final decision about inclusion. The study authors independently performed quality appraisal on each of the included papers, then met to compare ratings and discuss any discrepancies. Pairs of raters, using a structured appraisal tool [46] and its interpretation guide (Table 1), conducted the data extraction and review process. Kappa correlation was used to define the agreement prior to consensus for individual items, and intraclass correlation coefficient (ICC) was used to assess reliability between the raters, by SPSS™, version 20.0. This systematic review was not registered on PROSPERO but was reported in accordance with the Preferred Reporting Items for Systematic Reviews and Meta-analysis (PRISMA) statement (Additional file 1 Appendix) and COSMIN [42].

**Table 1:** Critical appraisal of study quality for psychometric articles: evaluation form [46]

Authors:	Year:	Rater:		
Evaluation Criteria		Score		
		2	1	0
Study question				
1. Was the relevant background work cited to define what is currently known about the measurement properties of measures under study, and the potential contributions of the current research question to inform that knowledge base?				
Study design				
2. Were appropriate inclusion/exclusion criteria defined?				
3. Were specific clinical measurement questions/hypotheses identified?				
4. Was an appropriate scope of measurement properties considered?				
5. Was an appropriate sample size used?				
6. Was appropriate retention/follow up obtained?(for studies involving retesting; otherwise n/a)				
Measurements				
7. Were specific descriptions provided of the measure under study and the method(s) used to administer it?				
8. Were standardized procedures used to administer all study measures in a manner that minimized potential sources of error/bias (including the study measure and its comparators?				
Analyses				
9. Were analyses conducted for each specific hypothesis or purpose?				
10. Were appropriate statistical tests performed to obtain point estimates of the measurement properties?				
11. Were appropriate ancillary analyses done to quantify the confidence in the estimates of the clinical measurement property (Precision/Confidence Intervals, benchmarks comparisons, standard error of measurement/ROC curves, alternate forms of analysis like SEM/MID,etc)?				
Recommendations				
12. Were clear, specific and accurate conclusions made about the clinical measurement properties; that were associated with appropriate clinical measurement recommendations and supported by the study objectives, analysis, and results?				
Subtotals(columns 1 and 2)				
Total score % (sum of subtotal/24 × 100), or, if for a specific paper or topic an item is deemed inappropriate, then you can sum of items, divide by 2 times the number of items, and multiply by 100 to get the percentage score				

©MacDermid 2011

## Results

Eighty full papers were selected for initial evaluation. At that phase, 64 papers were also excluded because they did not address to nerve injury by trauma. So, sixteen papers were ultimately included for critical appraisal and data extraction. Descriptive syntheses of the study population and sample, as well as a description of the instrument and its examined psychometric properties, are summarized in Table 2.

**Table 2:** Summary of studies addressing psychometrics properties of the outcomes instruments for nerve repair

Study	*N*	Population	Instrument	Psychometrics properties
Jerosch-Herold [47]	14	14 median nerve injuries	Set of sensory tests: S2PD, M2PD, LOCAL, Modified Pickup test Moberg, Object recognition, ADL	Reliability, validity
Brandsma et al. [60]	28	Leprosy disease and nerve injuries	Manual muscle strength test for specific movements related to hand intrinsic muscles	Reliability: inter/intraobserver
Rosén [61]	25	15 median, 10 ulnar nerve injuries	Set of tests: sensory function /perception of touch/vibration: Semmes-Weistein Monofilaments, vibrations of 30 and 256 Hz; Tactile gnosis: localization of constant and moving touch, S2PD, M2PD, shape-identification test; motor function tests: dynamometer; integrated sensory and motor function tests: Modified Pickup test Moberg, Sollerman grip test; cold intolerance test and hypersensitivity (qualitative); neurophysiological tests: electroneurography, electromyography	Construct validity
Rosén Lundborg [55]	54	26 median, 19 ulnar, 7 combined	STI test ™	Reliability: test-retest, internal consistency, validity
Rosén, Jerosh-Herold [59]	32	32 median and/or ulnar	S2PD, STI test ™	Responsiveness
Rosén, Lundborg [24]	70	34 median, 27 ulnar, 9 combined	Rosén-Lundborg score (sensory, motor and pain/discomfort domains)	Reliability: test-retest, internal consistency, construct validity (concurrent)
Jerosch-Herold [57]	115	23 median and ulnar nerve injuries	Set of sensory tests: WEST, S2PD, M2PD, LOCAL, modified pickup test Moberg, object recognition	Responsiveness
Rosén [56]	91	35 median, 23 ulnar	STI test™	Reliability: inter-tester
Schreuders et al. [48]	27	11 median, 9 ulnar, 7 combined	RIMH™	Reliability
Jerosch-Herold et al. [54]	39	23 median and 16 ulnar nerve injuries	Locognosia test	Reliability: test-retest, construct validity (discriminant)
Carlsson et al. [49]	159	54 nerve injuries	3 cold sensitivity questionnaires: CISS,CSS,PWES	Reliability: test-retest, internal consistency, content and construct validity
Dias et al. [50]	100	26 nerve injuries(1 ulnar, 25 median)	3 questionnaires: PEM, MHQ, DASH	Reliability: reproducibility/test-retest, internal consistency, construct validity
Naidu et al. [52]	94 hand and wrist problems	1 nerve injury	HAT, DASH, SF12	Reliability: test-retest, internal consistency, construct validity (convergent and discriminant)
Packham, MacDermid [53]	264	14.4% nerve injury/repair	PRWHE and subscales	Test-retest reliability and content
Chen et al. [51]	300	7 median, 6 ulnar, 9 radial nerve	DASH-CHNPLAGH	Translation and cultural adaptation, test-rest reliability and construct validity
Hsu et al. [58]	30	04 median, 11 digital nerve	MTT, SWM, S2PD, M2PD	Concurrent validity

*CISS* Cold Intolerance Symptom Severity, *CSS* Cold Sensitivity Severity, *PWES* Potential Work Exposure Scale, *PEM* Patient Evaluation Measure, *MHQ* Michigan Hand Outcome Questionnaire, *DASH* disabilities of the arm, shoulder and hand, *DASH-CHNPLAGH* simple Chinese version of DASH, *PRWHE* Patient-Rated Wrist and Hand Evaluation, *S2PD* static two-point discrimination, *M2PD* moving two-point discrimination, *LOCAL* area localization, *ADL* activities of daily living, *WEST* touch threshold test, *HAT* hand assessment tool, *SF12* Health Survey (Short Form 12), *STI test™* Shape and Texture Identification test, *RIMH*™ Rotterdam Intrinsic Hand Myometer, *MTT* manual tactile test

Data extraction was performed based on the MacDermid’s Guide of Critical Appraisal of Study Quality for Psychometric Articles [46]. Overall, the quality of each paper was variable, ranging from 11 to 22 out of maximum 24, although most of them (15) reached or exceeded at least 50% on the quality score rating (Table 3). Weighted kappa was calculated by a pre-consensus inter-rater agreement method on the 12 individual items of critical appraisal [46] ranging from 0.31 to 0.82. The ICC of total scores was 0.81, ranging from 0.38 to 0.94 between the two raters.

**Table 3:** Results of quality of studies on the psychometric properties of instruments for sensory and motor assessment after nerve repair based on “Critical Appraisal of Study Quality for Psychometric Articles: Evaluation Form” [46]

Study	1	2	3	4	5	6	7	8	9	10	11	12	Total	Percent
Jerosch-Herold [47]	2	2	0	1	0	NA	2	1	1	1	0	1	11	45.8
Brandsma et al. [60]	1	1	1	1	0	2	2	2	2	2	0	1	15	62.5
Rosén [61]	2	2	0	1	0	NA	2	2	2	2	0	2	15	62.5
Rosén and Lundborg [55]	2	2	2	2	0	2	2	2	2	1	2	2	21	87.5
Rosén and Jerosh-Herold [59]	2	2	2	0	0	2	1	2	2	2	1	2	18	75
Rosén and Lundborg [24]	2	1	0	1	0	NA	2	1	1	1	1	2	12	50
Jerosch-Herold [57]	1	2	0	0	0	1	2	2	2	2	1	2	15	62.5
Rosén [56]	2	1	2	0	0	NA	1	1	2	2	2	2	15	62.5
Schreuders et al. [48]	2	1	0	0	0	NA	2	2	2	2	0	2	13	54.2
Jerosch-Herold et al. [54]	2	1	2	1	0	NA	2	2	2	1	2	2	17	70.8
Carlsson et al. [49]	2	2	2	2	0	1	1	1	2	1	1	2	17	70.8
Dias et al. [50]	1	2	0	2	0	2	1	1	2	1	2	1	15	62.5
Naidu et al. [52]	1	2	0	2	0	1	2	2	2	1	1	2	16	66.7
Packham and MacDermid [53]	2	1	2	2	2	NA	2	2	2	2	2	2	21	95.4
Chen et al. [51]	2	1	1	2	2	2	2	2	2	2	2	2	22	91.6
Hsu et al. [58]	1	1	1	1	1	1	2	2	1	2	0	1	14	58.3

Item evaluation criteria of critical appraisal tool. *NA* not applicable

### Reliability

Reliability was the most frequently reported psychometric property for instruments that assessed muscle strength, sensory tests, functional tests, and patient-report questionnaires after nerve repair.

Manual strength test for nerve repair and leprosy disease had an intra-examiner Cohen’s weighted Kappa between 0.71 and 0.96 and inter-examiner of 0.72 to 0.93 [47]. A dynamometer specially designed for intrinsic muscle assessment had intraclass coefficient (ICC) index between 0.94 and 0.98 and SEM (standard error of measurement) between 2.2 and 5.8 [48]. The patient-reported outcomes measurements (PROMS), Cold Intolerance Symptom Severity (CISS), Cold Sensitivity Severity (CSS), Potential Work Exposure Scale (PWES) [49], Patient Evaluation Measure (PEM) [50], Michigan Hand Outcome Questionnaire (MHQ) [50], disabilities of the arm, shoulder and hand (DASH) [50, 51], hand assessment tool (HAT), Health Survey (Short Form 12) (SF12) [52], Patient-Rated Wrist and Hand Evaluation (PRWHE) [53], and the simplified Chinese version of DASH (DASH-CHNPLAGH) [51] had high internal consistency ranging from 0.88 to 0.96 with higher value for DASH 0.98 [50]. PRWHE was analyzed by Rasch analysis and supports internal consistency of the scale (*α* = 0.96) and reliability (as measured by the person separation index) of 0.95. The analysis on this paper supported a three-subscale structure (pain, specific activities, and usual activities) rather than the current divisions of pain and disability for this questionnaire, based on item response theory rather than classical test theory [53]. The CISS, CSS, and PWES PROMS had ICC of 0.85 [49]. A battery of sensory tests had moderate to high Pearson correlation coefficient reliability index (− 0. 47–0.90) [47]. The locognosia test had high test-retest ICC for median (0.92) and ulnar (0.85) nerve [54]. STI test™ (Shape and Texture Identification test) was shown to have a test-retest weighted Kappa value of 0.79, an internal consistency Cronbach’s alpha of 0.78 [55], and an inter-tester score of 0.66 [56].

### Validity

The cold sensitivity PROM had a Spearman’s correlation of 0.73 for CISS and 0.67 for CSS for construct validity analysis, and for content validity, it was found that 92% of patients answered the questionnaires [49]. The PEM, MHQ, and DASH PROMS had a Pearson’s correlation coefficient of > 0.38 [50]. Naidu et al. [52] analyzed 94 injuries, only one of which was a nerve repair, and reported 0.89 for Pearson’s correlation (construct validity). Chen et al. [51] correlated the Chinese version of DASH with SF-36 items and showed a negative correlation and positive correlation with a visual analogue scale (VAS).

The Pearson’s correlation was moderate to high (*r* = 0.90–0.47) for a battery of sensory tests [47]. The locognosia test had an effect size of 1.2 for median and 1.3 for ulnar nerve [57]. STI test™ presented sensitivity of 1.0 and specificity of 0.90 [55]. The Rosén-Lundborg score [24] had good to excellent results for its sub-domains for Spearman rank correlations. Manual tactile test (MTT) designed to assess functional aspects of Carpal tunnel syndrome including barognosis, stereognosis, and roughness discrimination tests had moderate concurrent validity for early sensory functional results in a nerve repair sample [58].

### Responsiveness

Responsiveness was tested only in two studies [57, 59]. Jerosch-Herold [57] analyzed in a battery of tests the standard response mean (SRM) and effect size and found the WEST™ to be the most responsive sensibility test (SRM = 2.4, effect size = 1.2) and also found that the 2PD (two-point discrimination) test was less responsive (SRM = 0.4, effect size = 0.1). Rosén and Jerosh-Herold [59] reported a SRM of 0.73 and a flooring effect for the STI test™ in relation to 2PD comparing patients in baseline and after 6 months of nerve repair.

## Discussion

Clinical measurement related to motor and sensory assessment after nerve repair was found to have excellent reliability but not sufficient evidence in terms of validity or responsiveness.

Reliability was most commonly assessed using test-retest [47, 49, 50, 52, 54, 55, 60]. A lesser number addressed inter-tester [55], intra-tester [60], and inter-instrument [48] reliability. All the papers that included internal consistency in their analyses linked this psychometric property to reliability and not to validity [55, 49, 50, 52, 53]. This is a common practice; although since internal consistency reflects the correlation between items, it is related to structural validity.

Validity was analyzed using content [55, 49], construct [47, 55, 48–52, 55, 61], and concurrent [58] approaches for assessment. Criterion validity was assessed by Rosén and Lundborg [55] for the STI test™. Only one study presented data of a PROM based on item response theory, rather than classical test theory [53].

Responsiveness was tested in only two studies [59, 57] addressing nerve repair which found static 2PD to be the least responsive. Although simple and easily applicable and used as reference to the modified highest classification [62] in many digital nerve repair studies [63, 64], this device has been criticized as having a low standardization as a tactile gnosis test without a performance protocol description present [62, 65].

The most common limitation found in this review was a lack of sample size calculation and a small amount of specific nerve repair volunteers inside the total sample in the studies included. As well, few studies described the tests, examiners, and procedures adequately. Since these are critical to fidelity of the assessment techniques, this can affect implementation of standardized methods in practice.

Two reviewers performed the selection of papers and critical appraisal. The other reviewers checked all the decisions. However, there were difficulties to identify and select the studies based on the samples, which should fulfill the inclusion criteria strictly related to nerve repair after trauma.

Clear presentation of clinical measurement objectives was often absent in the studies, which would benefit from better structure in terms of design and integration of methods, results, and discussion. Exceptions to those were the studies that assessed reliability and validity such as Carlsson et al. [49] who analyzed three different cold sensitivity PROMS questionnaires, Dias et al. [50] who compared three questionnaires for hand trauma including nerves, Jerosh-Herold [54] who analyzed median nerve repair in a battery of sensory tests, and Jerosh-Herold et al. [54] in other study that found excellent reliability and validity for locognosia test in peripheral nerve injuries of the hand.

Rosén and Lundborg [55] tested the STI test™ as a new tactile gnosis instrument; Naidu et al. [52] developed a PROM for patients with injuries of hand and wrist including nerve injury. Rosén and Lundborg [24] developed and described the model instrument for the documentation of outcome after nerve repair. These represent the newest dedicated outcome measures for nerve repair. While they are promising, the number of studies examining them is insufficient [65–67]. Future studies validating these tools are needed.

Two main forms of assessment of motor function were found in the selected studies: one involving the manual function muscle test [60] and the other dynamometers specially developed to assess the intrinsic muscles of the hand [48]. Brandsma et al. [60] analyzed the well-known manual muscle strength test focused on movement of hand intrinsic muscles in leprosy and nerve injuries patients and found excellent inter- and intra-observer reliability. Schreuders et al. [48] analyzed the Rotterdam Intrinsic Hand Myometer (RIHM™) to assess isometric intrinsic muscle strength for the hand and found excellent reliability. Manual muscle testing and dynamometers are frequently used by therapists and surgeon members of the Hand Societies of Surgery and Therapy throughout the world, for muscle testing [23, 68–71], but few have studied specifically patients with nerve injury and repair [70]. Few muscle strength studies presented data related to the quantification of intrinsic hand muscles, which measures motor dysfunction directly related to the median and ulnar nerve repair in the hand. Xu et al. [71] presented the Peg Restrained Intrinsic Muscle Evaluator (PRIME) but only for children. Normative results indicated that gender and age were significant predictors of strength and the device was considered a reliable tool for the quantification of intrinsic hand muscle strength in children. Jacquemin et al. [72] analyzed hand intrinsic muscle strength in relation to spinal cord injury (SCI) and other myelopathies to allow early diagnosis of neurologic decline. They used a handheld myometer in healthy volunteers and patients with SCI and found good inter-rater reliability. Bohannon and Andrews also found good inter-rater reliability of a handheld dynamometer testing procedure for neurologically involved patients with different conditions including hand muscles [73].

Shieh et al. [74] discussed the impact of nerve injury on sensorimotor control by exploring the effects of nerve regeneration on the control of pinch force in executing functional tasks in patients with median nerve repairs. The results revealed significant differences in the parameters of peak pinch force, baseline pinch force, force ratio, and the percentage of maximal pinch force output at different points in the course of nerve regeneration.

Callahan [69] classified the sensibility assessment for nerve lacerations into four categories: threshold test, functional tests, objective tests, and provocative tests. Threshold tests included touch-pressure threshold testing such as Semmes-Weinstein monofilaments (SWM) [16] to determine the minimum stimulus perceived by the patient and by measuring sensory impairment [69]. Functional tests included assessments of sensibility and disability, which were caused by sensory impairment and are considered integrative tests because they require higher levels of sensory processing than the thresholds [69]. The glabrous skin of the hand has sensory receptors, which allow the perception of sensory stimuli from periphery to central nervous system. A reliable and valid battery of tests must assess this perception in a different way for compression and following nerve injury, considering the characteristics of each measurement [22]. We found in this systematic review four studies [47, 54, 57, 61] that analyzed a battery of tests to find reliability, validity, and responsiveness of the instruments commonly used by therapists in clinical practice to assess functional outcomes including sensibility after peripheral nerve injury and repair in the hand. The quality of these studies based on the critical appraisal tool [46] was between 45.8 and 70.8% (scores 11–17). These studies suggested that reliable and valid assessment of re-innervation could be determined using SWM, and static 2PD for the tactile gnosis assessment [61]. Jerosch-Herold in 1993 [47] tested also a battery of tests in median nerve injuries but was unable to recommend specific sensibility tests that could be valid and suggested the inclusion of functional assessment for these patients. Jerosch-Herold in 2003 [57] found the WEST to have the highest responsive and the 2PD to be the least responsive in a battery of tests. Jerosch-Herold et al. in 2006 [54] analyzed locognosia through a standard protocol using SWM based on a localization hand chart divided in zones, first described by Winn-Parry, with ratio of scores between 0 and 10 (2 for correct localization, 1 for immediately adjacent, and otherwise zero). They found excellent test-retest reliability (ICC 0.92 for median and ICC 0.85 for ulnar nerve repair patients) and good construct validity (discriminant) based on the magnitude of difference between affected and unaffected hand (11.1 for median and 4.7 for ulnar nerve patients) with effect size respectively 1.2 and 1.3.

The STI test™ is an instrument developed by a research group in Sweden to assess tactile gnosis or the ability to identify shapes and textures without vision [55]. The developers found good test-retest reliability [55], good inter-tester reliability [56], and excellent criterion validity [55] related to this device for nerve injuries. The responsiveness was also good [59] and concluded that it could be used as an alternative to 2PD test. The MTT test was validated for a sample with nerve repair, although at an initial nerve regeneration phase [61]. As a sensory discriminative test, better responses usually will only occur after 6 months, in the dependency of axon regeneration and reinnervation on sensory receptors.

This review did not found any PROM specially developed for nerve repair outcome assessment. The HAT questionnaire developed by Naidu et al. [52] has been shown to be reliable and to measure the limitations of the hand and wrist after trauma and correlated well with the DASH and SF12 questionnaires, but it was validated for a small sample size which included only one case of nerve repair. Therefore, generalizations cannot be made based on those findings. Dias et al. [50] compared the reliability, validity, and ease of use of three PROMS: the PEM (Patient Evaluation Measure), the MHQ (Michigan Hand Outcome Questionnaire), and the DASH (disabilities of the arm, shoulder and hand) for hand disorders in which the sample included median and ulnar nerve repair. All were considered reliable, and their findings suggested that the PEM was the easiest to use; however, they could not confirm any kind of validity.

Van de Ven-Stevens et al. [75] reviewed the literature on the clinimetric properties of 23 instruments to asses hand’s activity limitation, based on the relevance of hand injuries. They analyzed reliability, validity, and responsiveness, as in functional tests and as in PROMS. Results from this study found that only five instruments adequately described the psychometric properties, but none of them had a positive rating. Galanakos et al. [76] in their systematic review highlighted the challenges in developing a clinical protocol based on a valid, reliable, and responsive prognostic model, that allow more effective determination of which patients have a better or diminished chance for a successful motor and sensory recovery after median or ulnar nerve injury on the upper extremity.

Nerve injuries cause pain, dysthesias, and cold intolerance symptoms that combined with impairments in motor and sensory function, which contribute to loss of hand function. The model instrument for documentation after nerve repair is described by Rosén and Lundborg [24] as an instrument which represents a combination of selected items grouped in motor (motor innervation and grip strength), sensory (sensory innervation, tactile gnosis, and finger dexterity) and pain and discomfort (hyperesthesia and cold intolerance) subdomains that together get a score for the peripheral nerve, ranging from 0 to 3. It is an effort to combine functional outcomes following nerve repair in a visual and quantitative way, developed essentially for median and ulnar nerve repair. While preliminary evidence supports multiple measures, the number, quality, and scope of current literature are insufficient to strongly recommend any specific strategy. However, given the conceptual specificity and early measurement properties, we recommend the use of the Rosén and Lundborg scale [24] for further validation and inclusion in nerve repair outcomes research.

Few studies in this review addressed the development of specific new PROMS or instruments based on COSMIN [77] to assess impairment and dysfunction related to nerve injury linked to a broader perspective, based on the International Classification of Functioning, Disability and Health (ICF). This classification, in a clinical context, could link the body functions and structures as motor and sensory domains, and the activity and participation as function domain through patient perspective by patient-rated outcome measures [34, 35].

PROMS that measures disability with subsequent validation analysis should be included in future nerve repair studies related to functional outcomes.

## Conclusion

For the motor domain, both manual muscle test and a dynamometer specially developed to measure strength of intrinsic muscles of the hand had excellent reliability but were not tested for validity and responsiveness in nerve repair.

For sensory domain assessment, the SWM was the most and 2PD the least responsive.

A battery of tests is suggested as more reliable and valid for nerve repair assessment.

Locognosia test seems to be valid, and the STI test™ is a valid and reliable instrument to assess tactile gnosis after nerve repair.

The model instrument for documentation after nerve repair has been shown to be valid and reliable as a quantitative tool to score nerve repair.

None of the PROMS (PEM, DASH, MHQ, and PRWHE) were considered valid for nerve repair, despite good reliability. In terms of cold sensitivity, three PROMS were considered reliable and valid to assess cold intolerance in nerve repair.

## Additional file

Additional file 1: Appendix: PRISMA checklist. (DOC 62 kb)

## Abbreviations

ADL: Activities of daily living
CISS: Cold Intolerance Symptom Severity
CSS: Cold Sensitivity Severity
DASH: Disabilities of the arm, shoulder and hand
DASH-CHNPLAGH: Simple Chinese version of DASH
HAT: Hand assessment tool
ICC: Intraclass correlation coefficient
LOCAL: Area localization
M2PD: Moving two-point discrimination
MHQ: Michigan Hand Outcome Questionnaire
MTT: Manual tactile test
PEM: Patient Evaluation Measure
PRISMA: Preferred Reporting Items for Systematic Reviews and Meta-analysis
PROMS: Patient-reported outcomes measurements
PRWHE: Patient-Rated Wrist and Hand Evaluation
PWES: Potential Work Exposure Scale
RIMH™: Rotterdam Intrinsic Hand Myometer
S2PD: Static two-point discrimination
SF12: Health Survey (Short Form 12)
STI test™: Shape and Texture Identification test
WEST: Touch threshold test
